# A Review of Fetal Bovine Serum in the Culture of Mesenchymal Stromal Cells and Potential Alternatives for Veterinary Medicine

**DOI:** 10.3389/fvets.2022.859025

**Published:** 2022-05-03

**Authors:** Cara R. Pilgrim, Kiera A. McCahill, Jenna G. Rops, Jaustin M. Dufour, Keith A. Russell, Thomas G. Koch

**Affiliations:** Department of Biomedical Science, Ontario Veterinary College, University of Guelph, Guelph, ON, Canada

**Keywords:** mesenchymal stromal cell, fetal bovine serum, platelet lysate, serum-free media, serum reduction, species specific serum

## Abstract

Fetal bovine serum (FBS) remains widely used as a supplement in cell culture media used in the isolation and expansion of mesenchymal stromal cells (MSC) despite longstanding practical, clinical, and ethical concerns over its use. As a result, research on alternative culture media supplement solutions that conserve crucial MSC characteristics has become increasingly relevant. Species-specific supplements and serum-free media such as platelet lysate or chemically defined media have been assessed for their effect in MSC cultures regarding proliferation, differentiation, and immunomodulatory capacity. While none of the alternatives offer a complete solution in replacing traditional FBS supplemented media for culturing MSCs for all species, short-term or transitional use of FBS-free media can perform equally well and could address some of the concerns over the use of FBS.

## Introduction

The field of stem cell research has gained significant traction due to the therapeutic potential of stem cells in human and veterinary medicine (1). Mesenchymal stromal cells (MSC) are a common type of multipotent cell used in experimental therapy for their capacity to differentiate into tissues such as cartilage and connective tissue and to modulate immune cell function (2, 3). These non-specialized cells can be obtained from many sources in the body and possess an extensive capacity to proliferate and self-renew making them the focus of potential treatments for many diseases including cardiovascular disease, spinal cord injuries, bone fractures, and autoimmune diseases (4, 5). However, the proliferation of these cells to useful therapeutic doses requires extensive culture in media which, often in the veterinary field, contains fetal bovine serum (FBS).

FBS is currently the standard culture supplement used for the expansion of domestic animal MSCs and most other types of cells grown in culture (6). It has been an integral part of general cell culture practices for more than 50 years due to its exceptional composition of factors required for both animal and human cell growth and proliferation (7). FBS also possesses very low levels of immunoglobulins relative to serum from mature cows, which reduces risk of provoking an immune response (8). However, FBS remains a concern to researchers and clinicians alike due to high rates of product variability, clinical risks of adverse reactions due to bovine proteins or disease, and ethical considerations over its production (9). Research into FBS alternatives for culturing MSC is therefore a very active area of investigation. In this review, the main concerns associated with culturing MSCs in FBS and the current state of FBS alternatives are discussed with a focus on work done in domestic animals.

## FBS in MSC Culture

Over 50 years ago, it was observed that FBS improved cell survival in culture with little understanding of how (10). Price and Gregory (11) identified key components of FBS including nutrients, hormones, transport proteins, growth factors, and attachment factors (Table 1), some combination of which support the attachment and growth of MSCs (12).

**Table 1:** Components of different media including FBS, PL, SFM, and DMEM.

**Fetal bovine serum**	**Platelet lysate**	**Serum free media (13)**	**DMEM (basal media)**
Adhesion molecules	Adhesion molecules	Adhesion molecules	Amino acids
Carbohydrates	Coagulation factors	Antioxidants	Glucose
Cytokines	Cytokines	Buffer	Inorganic salts
Essential amino acids	Growth factors	Carrier proteins	Sodium pyruvate
Growth factors	Protease inhibitors	Growth factors	Vitamins
Hormones		Hormones	
Non-protein nitrogen		Lipids	
Serum proteins		Polyamines	
Trace elements		Powdered medium	
Vitamins		Transport proteins	
		Vitamins	
		Water	

A critical property of MSCs for their isolation, identification, and culture is their adherence to plastic (14, 15). While there is yet to be a definitive factor in serum that promotes attachment of cells to plastic (15), it has been proposed that fibronectin and vitronectin both may contribute (16). A study of human MSCs showed that culture surfaces pre-treated with FBS promoted better attachment and proliferation than untreated culture surfaces (17). While in equine MSCs isolated from cord blood showed no difference in FBS coating on the culture flask on proliferation, while in bone marrow MSCs (BM-MSCs) fibronectin was required for cellular adhesion (18, 19). Yet, equine adipose derived MSCs (AD-MSCs) have been successfully grown without coating of the flask (20). In addition, studies growing MSCs in the absence of serum indicate that FBS supplementation is required for initial attachment during the isolation stage (15, 21).

The contribution to cell proliferation of various hormones, nutrients, growth factors, and other components in FBS is poorly understood. Some have suggested that hormones were the main avenue by which FBS supported cell growth (21). Through work done by Hayashi and Sato (21), FBS was replaced by supplementing media with T3, thyrotropin releasing hormone, transferrin, and parathyroid hormone, which successfully maintained the growth of a rat pituitary cell line for 10 days. This culture condition produced cellular proliferation at a rate of 60–100% that of FBS cultured cells, suggesting that these components of FBS are critical in supporting cell growth (21).

Glucose is one of the better understood molecules in MSC survival, and it serves as an example of why proper supplementation is key to MSC function. Nuschke et al. (22) found that human BM-MSCs rely heavily on glucose, as this key nutrient is rapidly depleted, while other nutrients, such as L-glutamine, pyruvate, and amino acids important for the growth of other cell types, are used sparingly (22). Glucose supply is important across all MSC source species and tissues since they possess very limited intracellular glucose storage or ATP reserve. Therefore, as little as 3–5 days of glucose deprivation in culture induces rapid cell death (22–25). However, high glucose concentrations have been associated with reduced proliferation, increased apoptosis, lower cell viability, and altered differentiation potential (26–28).

Growth factors are also important for MSC proliferation. Devireddy et al. found fibroblast growth factor (FGF), platelet derived growth factor (PDGF), transforming growth factor-beta 1 (TGF-ß1), and epidermal growth factor (EGF) all beneficial in supporting canine MSC growth (13). Proliferation of human BM-MSCs was greatly enhanced by TGF-ß and bFGF supplementation in the absence of serum, while TGF-ß alone slowed proliferation (15). Similarly, TGF-ß supplemented media in the absence of serum alone resulted in fewer canine AD-MSCs, but proliferation improved when additional growth factors like bFGF or PDGF were added (13). In equine cells, TGF-ß addition to FBS media increased proliferation rates when compared to plain FBS-supplemented media, likely due to serum providing other growth factors like PDGF and bFGF (29). These results illustrate the complex influences of growth factors required for cellular growth. More work is needed to identify the specific factors that FBS provides for MSC culture across different species.

### Challenges With FBS in MSC Culture

#### Variability

It is well-known among researchers that much variability occurs between batches of FBS (9). Hence, FBS batch testing is common practice within labs, which is both time-consuming and expensive (9). An early study assessed various batches of FBS from a single supplier, finding wide variation in component concentrations, which in turn impacted cell proliferation between batches (11). Variability had been noted even at its earliest use in cell culture as Puck et al. observed not all FBS batches promoted cell growth equally (10). They noted anecdotally that higher quality batches of serum came in the summer and fall months, and batches in the winter were less effective at growing human skin cells (10). Later studies began to assess which media variations impact cell growth. Certain factors, such as high growth hormone and low endotoxin level, were associated with improved growth of human lymphoma and primate kidney cell lines with other factors being equal (11). These influential factors varied widely between the batches tested (endotoxin 0.000–10 ng/mL, growth hormone 18.7–51.6 ng/mL) (11). Although these studies were not done on MSCs, they are indicative of the variability that occurs among FBS batches.

The quality of FBS has also been shown to differ based on collection and processing methods (30). It was found that FBS collected by research protocols had a consistently improved cell growth compared to commercially available FBS (30). They noted large variations in FBS's ability to support cell growth in the commercially produced FBS (30). More recently, commercial FBS was compared to FBS produced at the Avicenna Research Institute, and it was found that the two sera were very similar in physiochemical properties as well as hemoglobin and endotoxin level (31). When assessing its ability to support cell culture it was found that the viability significantly differed in 3 out of 10 cell lines when comparing commercial FBS to Avicenna produced FBS (31). This emphasizes the variability that can occur in FBS production. Gstraunthaler et al. (32) stated that variability is driven by poor regulations on FBS production. What begins as a practical problem for the successful *in vitro* culture of MSCs can turn into commercial and clinical problems in that FBS batch-to-batch variability may also lead to an inconsistent cellular product over the long-term.

#### Immune Response

The most important clinical concern in using FBS lies in its potential to provoke adverse reactions from bovine xenoproteins or disease from viral or prion contamination following injection of FBS-grown MSCs (8). MSCs have been shown to incorporate FBS proteins, which may lead to an expression of antibodies post-injection (33). Two bovine xenoprotiens that have been identified to be able to incorporate into mammalian cells include N-glycolylneuraminic acid (Neu5Gc) and bovine apolipoprotein B-100 (33, 34). The incorporation of Neu5Gc has been shown to greatly reduced the therapeutic potential of MSC due to Neu5Gc antibodies targeting injected MSCs (33). Neu5Gc is naturally produced in most animals, therefore the reaction to this molecule is largely a human concern rather than a veterinary one (33). Yet, there are conflicting reports about whether European dog breeds produce Neu5Gc (35, 36). Neu5Gc is not the only immunogenic contaminant of concern.

When children with osteogenesis imperfecta were injected with BM-MSCs, it was found that one of the six patients experienced an immune response against their MSC injection (37). This patient had developed antibodies against FBS with a 150-fold increase in these antibodies after their second injection suggesting that multiple injections can exacerbate immune responses (37). Another study looked at hematopoietic stem cell transplantation and immune response against the MSCs used and found that patients did not develop antibodies against MSCs, but anti-FBS antibodies were present (38). The expression of anti-FBS antibodies varied in the patients, consistent with healthy individuals (38). Additionally, Spees et al. observed that rat BM-MSCs cultured in typical 20% FBS media, had a humoral immune response present after repeated injection (3). Human BM-MSCs cultured in these conditions had 7 to 30 mg of bovine protein contamination per 100 million MSCs (3). Researchers transferred the human MSCs to media containing 10% adult human serum including growth factors for 5–10 days, resulting in elimination of 99.99% of FBS contamination from the BM-MSCs (3). When done in rat cells using adult rat serum with growth hormones, it eliminated the immune response previously observed with cells grown in FBS (3).

In an equine study, 89% of horses had anti-BSA antibodies prior to MSC administration most likely due to previous vaccinations, which may have also used FBS (39). It was observed that after administration of allogeneic adipose and bone marrow derived MSCs cultured in 10% FBS, there were no significant changes in their anti-BSA antibody titers (39). There was however the development of anti-MSC antibodies observed, but there was no correlation between the horses' level of anti-MSC antibodies and adverse reactions to the treatment, despite multiple MSC injections (39). Horses were also tested for hypersensitivity reactions after previous injection of MSCs cultured in FBS (40). Horses were intradermally injected with FBS, and this produced a significantly greater immune response than injection with saline (40).

A recent study sought to delineate the difference in immune response of horses injected with BM-MSC that were grown in FBS verses autologous and allogenic bone marrow supernatant (41). This work is one of few that focuses on the immune response and clinical outcome of equine BM-MSCs cultured in FBS. There were no differences in antibodies against FBS between the FBS and bone marrow supplemented group, but cells cultured without FBS showed less edema at the site of injection and more MSCs present in the synovium where they were injected (41). They found greater cell death of MSCs cultured in FBS and attributed this to the antibodies against FBS that were already present in the horse, likely due to previous vaccinations (41).

MSCs themselves are not immunogenic and are known to modulate the immune system through paracrine factors (42). These immunomodulatory functions of MSCs are well-established, but it is unclear the role FBS plays in promoting or detracting from MSCs' immunomodulatory function (43). In antimicrobial research, it was observed that lower concentrations of FBS increased equine BM-MSCs' antimicrobial function (44). They attributed this to FBS's ability to bind, and as a result inactivate, antimicrobial peptides (44, 45). Work by Tang et al. has shown that antimicrobial peptides, TP4-A12I, A15I, become inactivated when bound to the albumin and α1-antitrypsin complex in fetal but not adult serum (45). More work needs to be done to determine the effect that FBS has on MSC immunomodulatory function.

Despite these concerns, it should be clearly stated that there are both human and equine MSC clinical products produced in FBS-media that have received market approval in Europe, Canada, New Zealand, Japan, and South Korea (46, 47). The two veterinary stem cell products currently approved by the European Medicines Agency, HorStem (EquiCord) and Arti-Cell Forte (Boehringer Ingelheim), are both composed of allogeneic equine MSCs grown in FBS. Arti-Cell Forte is grown in serum-free medium for the last 72 h prior to clinical use, which may reduce the bovine protein content significantly based on previous studies done showing 95% reduction in 48 h of alternate culture (3, 48).

#### Contamination

FBS has been cited as a source of viral and prion contamination of cell culture, which can cause infection in patients being treated with MSCs (49–51). Parviviruses, pestiviruses, prions, and bovine viral diarrhea virus (BVDV) are of great concern and therefore diligently screened for (51, 52). BVDV is of particular concern as a study found that the virus is still infectious after heating for 30 min at 56°C, a common sterilization method (53) and can infect non-bovine ungulate species (54). Currently, FBS is filtered and tested for viral contaminants through the Code of Federal Regulations Title 9 (9CFR) guidelines, which require that FBS be tested for 7 specific viruses (50, 55). However, researchers have analyzed commercial FBS batches and determined that the rate of contamination is much higher than what is being reported suggesting that current screening methods are not adequate (49, 56–58).

Mycoplasma is a common form of contamination for cell culture that can arise from various different sources including human contamination and contaminated animal supplements (59). In the 1960–1970s, animal products were a major source of contamination, although presently FBS is rarely the source of mycoplasma contamination (59). This has been shown by a more recent study assessing mycoplasma content in Columbian produced FBS where they found no contamination of mycoplasma (60). A recent study assessed gamma irradiated FBS produced in Argentina for mycoplasma and found that 14% of FBS samples contained mycoplasma DNA out of 124 samples (61). When cells were cultured in these FBS batches, none of the cells tested positive for mycoplasma infection (61). This indicates that, although mycoplasmas were present in certain FBS batches, gamma irradiation did a successful job of inactivating them and preventing infection of cell culture (61).

### Alternatives to FBS in MSC Culture

#### Species-Specific Serum

When looking for replacements for FBS supplementation in cell culture, the first logical option is to use a species-specific serum. Yet, little research in the veterinary field has been done on the topic. When utilizing different sources of serum, it is important to considered differences in composition. Varying effects have been reported on the effect of horse serum compared to FBS on various cell types (62–66) However, while some comparative analysis of components has shown differences between horse and bovine sera (63), more comprehensive characterization is still needed. In the human field, adult serum has been used to expand MSCs in culture and have shown mixed results with more long-term studies needed (3, 67–69). A study done in human BM-MSC found that using allogenic serum for expansion resulted in cell cycle arrest and death when compared to autologous serum after a single passage (68). With autologous serum, umbilical cord MSC (CB-MSC) proliferated at a rate that was comparable to cells grown with FBS supplementation (69). This was extended over 20 passages with no changes in morphology (69). Moreover, BM-MSC and CB-MSC cultured in 10% adult serum show a similar ability to differentiate and immunomodulate when compared to FBS culture (68, 69).

A challenge with species-specific serum is the quantity of blood required to expand large numbers of MSCs (70). Horses have been shown to tolerate up to 25% of their blood volume being drawn (71). In dogs, a study suggests that there is increased risk in removing more than 15% of a sedated dog's blood volume (72). While also taking animal size into account, this would create a greater challenge in obtaining substantial amounts of canine blood when compared to equine. Recent studies have looked at the impact of different serum sources on equine BM-MSC growth (73, 74). One study found that BM-MSCs cultured in species-specific serum had similar viability and morphology with FBS-grown cells having a shorter population doubling time (74). The other study compared autologous horse serum and standard horse serum to FBS and found that autologous serum was comparable to FBS where standard horse serum decreased BM-MSC proliferation (73). Chondrogenic differentiation in autologous serum showed similar outcomes in equine serum, though cells grown in FBS showed greater bactericidal activity compared to equine serum (74). These researchers also detected no difference between the autologous and allogenic equine serum used, which differs from some of the work done with human MSCs (68, 74). It should be noted Eydt et al. failed to culture cells from 2 out of 12 donors used in autologous serum raising some concern about its reliability as a supplement (73). More work is needed in this area to determine how species-specific serum can impact all therapeutic functions of MSCs.

#### Serum Free Alternatives

##### Platelet Lysate

Platelet lysate (PL) was originally tested as an alternative culture additive due to its crucial role in hemostasis and growth in mammalian species (75). Mammalian blood plasma contains cell growth factors such as platelet derived growth factor (PDGF), vascular endothelial growth factor (VEGF), and epidermal growth factor (EGF) (75). PL is prepared from the serum of pooled allogenic or autologous blood (76). This eliminates the risk of cross-species contamination, and pooled allogenic PL is inexpensive to produce and use in large animal cell cultures (76–78).

Studies have shown comparable proliferation rates of equine MSCs derived from adipose tissue, cord blood, and bone marrow in equine PL (ePL) when compared to FBS (76, 78–82). Some studies found a clear dose-dependent positive effect on the proliferation rate of equine MSC with increased concentrations of ePL (76, 78, 79). However, the dose-dependent effect of PL on MSC proliferation may be limited, and there may be an optimal threshold for ePL concentration as we have previously reported (78). ePL concentrations higher than 30% showed a detrimental effect on CB-MSC proliferation rates compared to equal concentrations of FBS (78). One study assessed chondrogenic differentiation in equine BM-MSCs cultured in ePL and found no difference between ePL and FBS cultured cells (80), while another study in equine peripheral blood derived MSCs found a decrease in proliferation in cells cultured in 15% PL compared to FBS (40). In canine AD-MSCs, Hagen et al. found that when cultured in PL, the cells lost their spindle-like formations and were unable to be dissociated from the culture flask (81). In our canine studies, we found that PL induced spontaneous adipogenic differentiation with lipid droplets appearing in as few as 4 days in BM-MSC and AD-MSCs (83). Another group of researchers did not observe the same spontaneous adipogenic induction from canine PL, but only cultured AD-MSCs for 3 days (84). Directed differentiation of canine and equine MSCs has been shown to be maintained in cells cultured in PL when compared to FBS (75, 82–84). Immunomodulatory function of MSCs also needs to be preserved in PL culture. Both Naskou et al. and Yaneselli et al. demonstrated that equine BM-MSC immunomodulatory properties were comparable between those cultured in PL and those in FBS (75, 76).

It is important to note that heparin is often added to PL culture to prevent gel formation in cell cultures (85). As heparin is usually porcine- or bovine-source, the resulting cell product will not be free of xenoproteins. If a xeno-free product is desired, mechanical or chemical depletion of fibrinogen in PL should be considered as an alternative (85, 86).

##### Chemically Defined Media

A defined serum-free media (SFM) represents an ideal formulation for consistency across all cell cultures, experiments, and labs (87). It provides standardized components of the media while mitigating issues of contamination, variability, and ethics (88). There are many chemically defined or semi-defined SFM products that are currently commercially available for cell culture (Table 2). However, these SFM are optimized for human stem cell culture. Researchers have been investigating if these SFM can be used with veterinary animal cell cultures (18, 91, 92).

**Table 2:** Autologous blood products evidence table.

**Paper**	**Hypothesis/objectives**	**Cell type/PL type**	**Parameters assessed**	**Findings/conclusions**
**Canine MSC**				
Russell et al. (83)	Investigate if PL is comparable to FBS in culturing Canine adipose and bone marrow MSC	Canine bone marrow MSC and adipose MSC	Isolation Proliferation Spontaneous Differentiation Directed Differentiation	MSC isolation was not successful in PL. Maximal proliferation occurred in 10% PL, Differences were noted in morphology after 21 days of culture in PL. Cells cultured in PL tended toward spontaneous adipogenic differentiation. PL cultured cells were able to undergo directed adipogenesis and chondrogenesis.
Lima et al. (84)	Assess PL effect on proliferation and differentiation of Canine Adipose-MSC.	Canine adipose MSC	Proliferation Differentiation	Cultured for 3 days in PL or FBS supplemented media. Found that 5 and 10% PL supported better cell proliferation than media supplemented with 10% FBS. Differentiation occurred in a 3D PL gel with chondrogenic medium.
Suelzu et al. (89)	Assess the ability to PL to support canine adipose MSC proliferation and differentiation.	Canine adipose MSC Allogenic and autologous PL	Proliferation Differentiation	Found that allogenic and autologous were able to support proliferation of canine AT-MSC (cultured for 72 h). Cells were successfully differentiated along adipogenic and osteogenic lines.
Hagen et al. (81)	Ability of PL to support canine MSCs	Canine adipose derived MSCs	Proliferation Apoptosis Differentiation	Canine MSCs loss spindle formations and were unable to fully dissociate from culture flask. Found no difference in differentiation. Showed less apoptosis when cultured in 10% PL compared to 2.5% PL.
**Equine MSC**				
Russell et al. (90)	Assess ability of PL to support Equine CB-MSC at various concentrations compared to FBS.	Equine cord blood MSC	Proliferation	No significant difference found in cells cultured in PL vs. FBS in a concentration up to 30%. At higher concentrations of PL, proliferation was negatively impacted.
Seo et al. (82)	Compare allogenic PL to FBS in the culturing of Equine bone marrow MSC.	Equine bone marrow MSC Autologous PL	Isolation Proliferation	Comparable isolation between PL and FBS. PL MSC were smaller, and more spindle shaped than FBS MSC. No difference in number of cells after 10 days or in population doubling time. PL MSC required less trypsinization to remove from the plate, suggesting weaker attachment.
Hagen et al. (77)	Produce a standard method of PL production. Asses the how PL effects the function of MSC compared to FBS.	Equine adipose-MSC Pooled PL	Proliferation Viability Directed differentiation	Cells grown in PL had a more rounded shape. No significant difference in proliferation between 10% PL and 10% FBS. Proliferation variable at 5% PL and insufficient at 2.5% PL. Adipogenic differentiation consistent across groups, osteogenesis was weaker in the 10% PL group. Cells cultured for 1 passage only.
Naskou et al. (75)	Assess how Equine BM- MSC respond to long term culture in equine PL.	Equine bone marrow MSC Pooled PL	Proliferation Viability Directed differentiation	Cells showed no significant difference in proliferation and viability when grown in PL compared to FBS (32 days of growing). PL and FBS showed no significant difference in osteogenic differentiation, but PL showed a significant improvement in chondrogenic differentiation.
Pezzanite et al. (74)	Comparing FBS to allogenic and autologous equine serum in sustaining BM-MSC in culture	Equine bone marrow MSC	Viability Proliferation Morphology Cytokine secretion Antimicrobial peptides Bactericidal activity Chondrogenic differentiation	FBS resulted in shorter population doubling time and greater secretion of cytokines and antimicrobial peptides. No difference was noted in chondrogenic differentiation.
Bue et al. (79)	Compare *in vitro* proliferation of equine MSC in PL vs. FBS	Equine adipose MSC	Proliferation	PL showed a dose-dependent positive effect on equine MSC proliferation at 24, 48 and 72 h.
Yaneselli et al. (76)	Determine if PL (basal and concentrated) grown MSCs will demonstrate similar immunophenotypes and other properties compared to MSCs grown in FBS.	Equine bone marrow MSCs	Immunophenotype Proliferation Cell viability (post-cryopreservation)	Proliferation significantly increased in concentrated PL media, viability comparable between concentrated PL and FBS, slight increase in Il-6 and COX-2 immunomodulatory gene expression in PL vs. FBS.
Eydt et al. (73)	Assess differences in standard horse serum and autologous equine serum compared to FBS	Equine bone marrow MSCs	Proliferation	Standard horse serum was found to have decreased proliferation compared to FBS. Autologous equine serum was found to be comparable to FBS. Two out of 12 cell lines cultured in autologous serum were not successful.
Chapman et al. (80)	Platelet lysate ability to culture equine BM-MSCs	Equine bone marrow MSCs	Proliferation Chondrogenic differentiation	Able to culture cells at passage 3, found no difference in proliferation or chondrogenic differentiation. It should be noted researchers were not able to create enough PL, so cells were cultured in FBS until passage 3 and switched to PL.
Longhini et al. (40)	Testing alternate mediums to FBS to culture peripheral blood MSCs	Equine peripheral blood derived MSCs	Proliferation Surface markers	Autologous serum was found to have similar proliferation to FBS, but changes in surface markers. PL was found to have decrease in proliferation but no changes in surface markers. Autologous heat inactivated serum supplemented with recombinant equine growth factors maintained proliferation and surface marker expression comparable with FBS.
Hagen et al. (81)	PL ability to culture equine MSCs	Equine adipose derived MSCs	Proliferation Differentiation	PL comparable to FBS in proliferation and differentiation.

The challenge with developing SFM is obtaining definable components that can support cell growth and function. Proliferation is one of the standard tests used to evaluate the efficacy of SFM culture (93). StemPro MSC SFM (Invitrogen), StemPro MSC SFM Xeno-free (Invitrogen), TheraPEAK MSC growth medium (LonzaBiosciences), MesenCult-XF (StemCell Technologies), BD-Mosaic hMSC SF (BD Biosciences), and Insulin-Transferrin-Selenium (ITS) (Gibco) (Table 3) all demonstrated slower doubling times for human BM-MSC, AD-MSC and MSCs derived from amniotic tissue compared to FBS (18, 87, 94, 95). In the veterinary field, Ultra-culture and UltroserG SFM demonstrated significantly reduced proliferative capacity in canine and equine AD-MSC, but significantly increased proliferation in porcine AD-MSC emphasizing the challenge of species-specific differences (1). Another group of researchers found no difference in proliferation of equine BM-MSC and canine AD-MSC grown in StemPro SFM when compared to FBS (18). Work done in our lab found that using StemPro SFM in equine CB-MSC culture resulted in slower proliferation compared to FBS (96). These differences could be attributed to tissue source variation. Finally, utilizing StemMACS MSC expansion kit, researchers were able to isolate and culture equine AD-MSCs with no difference in proliferation compared FBS cultured cells (20). Yet, these cells displayed altered morphology when visually assessed, and altered surface marker expression when compared to control cells (20).

**Table 3:** Commercially available SFM investigated in veterinary medicine.

**Product name**	**Intended application**	**Studies in vet medicine**
BD mosaic MSC serum-free (BD biosciences)	Human BM-MSC	None
Insulin-trasnferrin-selenium (Gibco)	Mammalian cells	None
MesenCult-XF (StemCell Technologies)	Human BM-MSC	None
NutriStem (Sartorius)	Human MSC	(97)
RoosterNourish-MSC-XF (Rooster Bio)	Human BM-MSC	(13)
StemMACS (Miltenyi Biotec)	Human BM-MSC	(20)
StemPro MSC SFM (Invitrogen)	Human MSC	(18, 96, 97)
TheraPEAK (LonzaBiosciences)	Spodoptera Frugiperda (Sf9) cells	None
UltraCULTURE (Lonza)	Mammalian cells (variety)	(1)

Osteogenic and chondrogenic differentiation potential of BM-MSC, AD-MSC, and amnion derived MSCs after culture in SFM, BD Mosaic hMSC SF, ITS, and StemPro SFM was comparable to cells cultured in FBS (87, 94, 95). Canine, equine, and porcine MSC osteogenic and chondrogenic differentiation potential was evaluated after culture in Ultra-culture and UltroserG SFM, and both were found to support capacities equal to FBS-cultured AD-MSCs (1). There is limited research evaluating veterinary animal MSC expansion in BD Mosaic hMSC SF, ITS, and StemPro SFM, which could be an area of interest moving forward.

Looking at immunomodulatory properties of mammalian MSC, StemPro MSC SFM produce altered immunomodulatory function of equine and canine BM-MSCs when compared to FBS-containing medium (18). It was observed that both equine BM-MSCs and canine AD-MSCs maintained their ability to inhibit T-cell proliferation, although MSC mediated IL-10 secretion was significantly increased and PGE2 secretion was significantly decreased in SFM (18).

In addition to those commercially available, researchers have attempted to formulate their own SFM using a species-specific approach. Devireddy et al. (13) formulated a SFM (Tables 1, 4) targeting canine AD-MSCs. While this medium did support proliferation of canine AD-MSCs, a challenge with this medium is that it is not completely free of xenogeneic proteins in that BSA was included, which may pose a risk of immune reaction in the recipient (13). More research needs to be performed to confirm the effects of different SFM formulae on MSCs from different species and tissue sources.

**Table 4:** Serum free media evidence table.

**Source**	**Purpose**	**Sample type/MSC origin**	**Experiments**	**Main results/conclusions**
**Human MSC**				
Gottipamula et al. (94)	Comparison of 5 commercially available SFM against FBS: StemPro MSC SFM, StemPro MSC SFM Xeno-free, TheraPEAK MSCGM-CD, Mesencult-XF and BD Mosaic MSC serum-free	Human bone marrow MSC	Proliferation Growth rate Morphology Differentiation Immunomodulation	Mesencult-XF and BD-SFM best supported MSC growth in comparison to the other SFM. Mesencult-XF was excluded from the “scale-up” study due to altered immunoregulatory presentation. BD-MSC supported proliferation without compromising functionality of MSC across other measures.
Lee et al. (87)	Compare FBS and SFM effects on MSC characteristics (SFM not stated)	Adipose MSC (species unspecified)	Proliferation Growth rate Cell viability Gene expression	SFM culture demonstrated faster population doubling times (proliferation and growth rate improved) compared to FBS. Cell viability and differentiation capacity were improved in SFM compared to FBS. Gene expression and mRNA stability supported previous observations.
Liu et al. (95)	Insulin-Transferrin-Selenium (ITS) SFM effective supplement for MSC culture.	Human amnion MSC	Proliferation Migration Differentiation Phenotype	Proliferation, migration, differentiation potential and cell phenotype were all maintained in ITS supplemented media.
**Animal MSC**				
Clark et al. (18)	HYPERflask and StemPro MSC SFM may alter immune function of MSC.	Equine bone marrow derived MSC Canine adipose derived MSC	Proliferation Phenotype Immunomodulation	HYPERflask—normal proliferation, phenotype and immunomodulatory function compared to FBS (HYPERflask used with FBS containing media) StemPro SFM—normal proliferation and phenotype, altered immunomodulation (Il-10 increased expression, PGE2 decreased expression).
Schwarz et al. (1)	Ultra-culture + UltroserG SFM and media supplement as effective culture conditions for multiple species MSC expansion.	Canine, equine and porcine adipose MSC	Proliferation Differentiation	Equine and canine MSC did not proliferate to the same capacity in SFM as FBS, differentiation capacity remained unaffected. Porcine MSC had increased proliferation rates in SFM than FBS and maintained differentiation capacity.
Devireddy et al. (13)	Unique Serum Free Media Formulation (in this table) to support the growth of canine MSC	Canine adipose MSC	Proliferation Isolation	Commercially available SFM (Rooster Bio) was not able to successfully support canine MSC. Developed unique serum free media that could support canine MSC (still contains xenoproteins).
Schubert et al. (20)	Evaluate suitability of SFM designed for human MSC for use equine MSC culture (StemMACS XF expansion kit)	Equine adipose MSC	Proliferation Cell morphology Differentiation Immunophenotype	Human MSC demonstrated more consistent growth in SFM as FBS, while equine MSC demonstrated altered surface immunophenotypes and proliferative capacity. Suggests that requirements for SFM culture conditions are species-specific.
Kuwahara et al. (97)	Can MSC be grown in SFM and can functional EV be collected from these cells.	Canine bone marrow MSC	Proliferation EV treatment	NutriStem XF media and StemPro was evaluated. No significant difference in growth rates between the two serum free media and FBS supplemented media. NutriStem had slightly altered morphology where they were more star-shaped compared to StemPro produced more spindles. EVs from StemPro media had significantly less IL-beta1. Were able to collect EV from MSC grown in SFM.

#### FBS Reduction

With the shortcomings of serum alternatives, it seems that complete replacement of FBS in MSC culture may not be feasible at present. It may prove beneficial to investigate ways that serum can be reduced until a better solution is found.

There is evidence to suggest that reducing use of FBS in MSC culture may be sufficient to overcome some of the major concerns related to its use. Currently, FBS is still required to isolate and establish MSC cell cultures. This results in the cells being switched from FBS to an alternative media after they are well-established (74). Clark et al.s' used commercially available SFM, but required the culture flasks to be coated in bovine fibronectin, a potentially xenogeneic substance (18). A short-term transition period just prior to clinical use may be advisable to produce a xenogeneic-free cellular product. This was shown in human BM-MSCs where media was switched from containing fluorescently labeled FBS to adult human serum for a 42-h culture period, after which signal from the FBS was reduced 10,000-fold (3). In a separate study, switching BM-MSCs from FBS medium to SFM found a 95% reduction in intracellular labeled FBS after 48 h (48). Similarly, there was a significant decrease in incorporated Neu5Gc from FBS when BM-MSCs were switched to media containing adult human serum for 1 week (33). In equine BM-MSCs, cells that were initially cultured in FBS then switched to equine serum for 72 h were found to be comparable to cells cultured only in FBS supplemented medium (74). Reducing FBS concentrations to 1–2% has also been shown to improve BM-MSCs' antimicrobial function with a similar trend seen with equine serum (44, 74). While some caution should be observed that a stress response may be caused due to the abrupt change in culture conditions, a short period of serum reduction or substitution may be a viable option to reduce risk of adverse outcomes from FBS.

## Conclusion

Overall, FBS is effective when it comes to promoting the establishment and expansion of MSCs *in vitro*. Yet, there are enough concerns associated with its use in cell therapy to merit a search for alternatives. Based on the current state of the literature, there does not seem to be a clear replacement for FBS for all aspects of its current use. Researchers have experimented with species-specific serum, serum-free, and serum-reduced media, but none provide a perfect replacement option. In the veterinary space, PL or a defined SFM may hold the most promise. Short-term PL culture appears to support proliferation, differentiation capacity, and immune function comparable to FBS culture. To minimize risk, using FBS-free media for a short-term transition culture period preceding clinical administration may be prudent.

## Author Contributions

CP conceptualized the study. CP, KM, JR, JD, KR, and TK outlined the paper collaboratively. CP, KM, JR, and JD reviewed the literature and wrote the report collaboratively. KR and TK supervised the work and edited the manuscript. All authors read and approved the final version of the manuscript.

## Funding

This work was partial supported by Mitacs Accelerate (Grant #460950) and the Department of Biomedical Sciences, Ontario Veterinary College.

## Conflict of Interest

The authors declare that the research was conducted in the absence of any commercial or financial relationships that could be construed as a potential conflict of interest.

## Publisher's Note

All claims expressed in this article are solely those of the authors and do not necessarily represent those of their affiliated organizations, or those of the publisher, the editors and the reviewers. Any product that may be evaluated in this article, or claim that may be made by its manufacturer, is not guaranteed or endorsed by the publisher.
